# The Many Faces of Primary EBV Infection: A Case Series of Its Diverse Presentations

**DOI:** 10.3390/jcm14248747

**Published:** 2025-12-10

**Authors:** Sylvia Lörcher, Christian Abegg, Alexander Wepf, Urs Karrer, Michael Osthoff

**Affiliations:** 1 Department of Internal Medicine, Cantonal Hospital Winterthur, 8401 Winterthur, Switzerland; 2 Institute of Laboratory Medicine, Cantonal Hospital Winterthur, 8401 Winterthur, Switzerland; 3 Division of Infectious Diseases and Hospital Epidemiology, Cantonal Hospital Winterthur, 8401 Winterthur, Switzerland; 4 Department of Clinical Research, University of Basel, 4001 Basel, Switzerland

**Keywords:** primary Epstein-Barr virus infection, cholestatic hepatitis, Hoagland sign, late seroconversion, hemophagocytic lymphohistiocytosis, mononucleosis

## Abstract

Epstein–Barr virus (EBV) is distributed worldwide and shows a seroprevalence of over 90% in adults, while seroprevalence in children varies depending on geographic and socioeconomic factors. Although primary EBV infection (pEBV) is often asymptomatic in early childhood, infection later in life may present with a variety of symptoms, most commonly as infectious mononucleosis, though many other clinical manifestations may occur. We present four clinical cases to illustrate the diverse and uncommon manifestations of pEBV and to support diagnostic reasoning. The first case demonstrates a diagnostic challenge of pEBV in a patient with severe cholestatic hepatitis in the setting of a recent travel. The second case highlights bilateral eyelid swelling (Hoagland sign) as a potentially isolated early symptom of pEBV, which clinicians should consider within its broad differential diagnosis. In the third case, we emphasize the importance of clinical judgment in contrast to premature closure in the face of repeatedly negative EBV serologies and advocate for further diagnostic evaluation, such as PCR testing, when pEBV is strongly suspected. The fourth case describes a fatal outcome of pEBV late in life, complicated by hemophagocytic lymphohistiocytosis. Unusual presentations of pEBV may complicate the diagnostic process and may lead to unnecessary testing. Our case series underscores the broad clinical spectrum of pEBV and highlights key features that aid in distinguishing it from important differential diagnoses. Awareness of characteristic laboratory findings, including reactive lymphocytosis, elevated large unstained cells, persistent fever, and lymphadenopathy, splenomegaly, as well as mild-to-moderate hepatitis is essential for guiding a targeted diagnostic approach for pEBV.

## 1. Introduction

Human Herpesvirus 4 (HHV-4), or Epstein–Barr Virus (EBV), infects over 90% of adults globally [1,2]. In children, seroprevalence varies (20–80%), influenced by geographic and socioeconomic factors [3]. In low-income countries, primary infection typically occurs in early childhood, while in high-income settings, there is a trend to delayed exposure. Consequently, seroprevalence is higher in children from lower socioeconomic backgrounds [4,5].

EBV spreads mainly via salivary contact. In young children, this occurs through close caregiving, while in adolescents, kissing is the primary route. Transmission through sexual contact, blood transfusions, or organ transplants may occur but play an epidemiologically minor role [3,6]. No evidence supports transplacental transmission [4].

EBV is a primate-specific gammaherpesvirus with strong tropism for B cells and oropharyngeal epithelium [2,4]. Following oral transmission, it causes lytic infection in the oropharynx and establishes latency in B cells by downregulating antigen expression. It has lifelong persistence and may reactivate during immunosuppression. Most primary infections, especially in children, are asymptomatic or mild [7]. In industrialized countries, delayed infection increases the risk of symptomatic disease. Infectious mononucleosis (IM), characterized by fever, fatigue, pharyngitis, and lymphadenopathy, is common in adolescents and young adults, affecting up to 75% after primary EBV infection [3]. Symptoms arise after an incubation period of 1–7 weeks and are the result of a pronounced immune response, particularly from CD8+ T-cell expansion [8] and not the virus itself. Complications include splenomegaly, hepatomegaly, rash, and, rarely, hemophagocytic syndrome (HLH) or splenic rupture [9]. EBV is also an oncogenic virus and is linked to several malignancies, including Burkitt’s lymphoma, Hodgkin’s lymphoma, nasopharyngeal carcinoma, and gastric carcinoma [2].

Acute EBV infection often presents with marked lymphocytosis, elevated liver enzymes, and inflammatory markers. Monospot tests detect heterophile antibodies during IM [10]. EBV-specific serological testing consists of IgM and IgG against the virus capsid antigen (VCA) complex. In our experience, the patients with acute EBV are often positive for VCA-IgM and -IgG, but single-positive VCA-IgM during the initial presentation is also possible. Both can be found positive during an acute infection due to the incubation time of 6 weeks [10]. EBV nuclear antigen 1 (EBNA-1) IgG appears 3–6 months post-infection and excludes acute primary infection if detected early [2]. Viral load is assessed via EBV PCR, and is particularly helpful for the diagnosis of reactivation in immunocompromised individuals and may be helpful if the serologic pattern remains inconclusive after repeat testing (Table 1).

Therapy is supportive, including acetaminophen or nonsteroidal anti-inflammatory drugs for fever and sore throat. Corticosteroids may be used in case of airway compromise and in severe, life-threatening cases to reduce immune-mediated symptoms (e.g., liver failure, severe immune-mediated anemia). There is insufficient evidence to recommend corticosteroids for symptom relief in general. Although acyclovir may reduce EBV replication, antivirals do not provide any clinical benefit apart from reducing oropharyngeal shedding [12,13]. In the case of HLH triggered by EBV, rituximab-containing chemotherapy regimen may be beneficial, in particular, if only B cells are infected [14,15].

Given the high global seroprevalence of EBV and the ongoing shift of primary infection to older age groups, we present four cases to illustrate the various clinical presentations of primary EBV infection and to highlight common features to aid in the clinical reasoning process and establishment of a diagnosis. The four cases were selected in the time period of March 2023 to November 2024 among adult patients (>18 years), which were discussed during a weekly grand round meeting leading up to the diagnosis of primary EBV infection.

## 2. Case Series

### 2.1. Case 1: Severe Cholestatic Hepatitis

A 21-year-old woman with jaundice was referred to our hospital. Two weeks earlier, she had returned from a 7-day trip to Greece. A week upon return, she developed high-grade fever and weakness followed by loss of appetite, nausea, and generalized pruritus. Her medication included liraglutid for weight loss and occasional paracetamol. Five days after symptom onset, outpatient laboratory tests showed a cholestatic hepatitis with liver enzymes elevated threefold. Abdominal ultrasound revealed normal hepatic architecture and bile ducts, but mild splenomegaly (16 cm, 481 mL). Mild lymphocytosis was noted. On admission, she had one palpable submandibular lymph node, and jaundice. Liver enzymes had increased to tenfold the upper limit (Table 2, and Supplemental Table S1).

Initially, a broad differential diagnosis was entertained by the treating team, including viral, toxic, ischemic and autoimmune hepatitis, and several tests were ordered such as serologies for EBV, cytomegalovirus (CMV), human immunodeficiency virus (HIV), Hepatitis A, B, and C, antinuclear antibodies (ANA), and ceruloplasmin.

Of note, serologies were negative for EBV VCA-IgG and EBNA-1-IgG, with a borderline EBV VCA-IgM test result. Given the reactive lymphocytosis, splenomegaly, and young age, an EBV polymerase chain reaction (PCR) was ordered and turned out positive. Subsequently, the EBV VCA-IgM was positive on repeat testing within 3 days, confirming primary EBV infection as the cause of the cholestatic hepatitis (Supplemental Table S1).

Retrospectively, her symptoms of fever and fatigue can be assigned to an IM. Typical symptoms for IM might be mild or reported only on specific questioning. Features of IM in the history and clinical examination in this case were present but received limited attention due to the relatively mild clinical presentation compared to the pronounced cholestatic hepatitis. Her recent travel history was also misleading and initially directed clinical suspicion toward other causes of cholestatic hepatitis. The clinical presentation of sudden onset of fever, cervical lymphadenopathy, lymphocytosis in the laboratory findings, and splenomegaly were indicative of an EBV-induced cholestatic hepatitis.

The hepatic injury is mediated by the host immune response rather than by direct viral cytopathic effects. It is predominantly characterized by lymphocytic infiltrate, consisting primarily of reactive cytotoxic (CD8) T lymphocytes within the portal tracts and sinusoids leading to inflammation, and if disruption of bile flow occurs, resulting in cholestasis [16].

### 2.2. Case 2: Bilateral Eyelid Swelling (Hoagland Sign)

A 24-year-old woman was referred with sudden onset of bilateral eyelid swelling and headache. The swelling had begun one day prior, the sinusitis-like, mainly periorbital headache three days earlier. Vision was not affected. No travel or known sick contacts were reported. Her work consisted of regular close contact with children. Besides a contraceptive, there was no regular medication.

**Figure 1:**
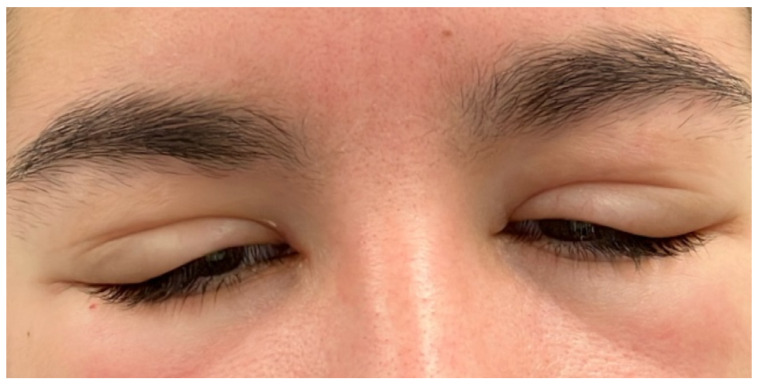
Upper eyelid swelling (Hoagland sign) in primary EBV infection.

On admission, bilateral eyelid swelling was noted without signs of local infection (Figure 1). Ear and oral examination was normal. Fever was not present. Laboratory tests revealed isolated lymphopenia (Table 3 and Supplemental Table S2). A head computer tomogram (CT) showed soft tissue swelling suggestive of cellulitis and bilateral cervical lymphadenopathy. Pansinusitis was ruled out. She was sent home on NSAIDs for symptom control.

She re-presented the next day with worsening eyelid swelling and sudden onset of fever (39 °C), without further new clinical or laboratory findings. Empiric treatment with amoxicillin/clavulanic acid for facial cellulitis was initiated. However, she had not improved on follow-up three days later. Antibiotics were changed to doxycycline for suspected atypical pathogens. Repeated laboratory tests revealed reactive lymphocytosis and mildly elevated transaminases. Initial EBV serology showed borderline VCA-IgM with negative VCA-IgG and EBNA-1. A week later, repeat testing confirmed primary EBV infection (positive VCA-IgM and IgG, negative EBNA-1). Asymptomatic hepatitis was attributed to EBV, and liver function tests peaked one week after referral (Table 3, and Supplemental Table S2).

This case was challenging as laboratory features of primary EBV infection only developed several days after the initial presentation, although bilateral eyelid swelling is a classical presentation of primary EBV infection. Supporting this diagnosis was the development of well-known clinical features of an IM, with sudden onset of fever, lymphocytosis and cervical lymph node enlargement (CT scan). Other differential diagnoses for upper eyelid edema include thyroid disease, lymphoma, allergic reaction, dermatitis, Parvovirus B19 infection, Kawasaki disease, sarcoidosis, trichinosis, and bilateral periorbital cellulitis [17]. Recently, bilateral eyelid swelling was also reported in the setting of intrahepatic cholangiocarcinoma [18].

Bilateral eyelid swelling, known as the Hoagland sign, was first described in 1952. It is thought to result from periorbital lymphocytic infiltration or lacrimal gland enlargement [19]. The sign typically appears early in the course of EBV infection and may precede classic features of IM as in our case, aiding early diagnosis of an EBV infection if being aware of this sign [17]. It is suggested that it occurs predominantly in adolescents and young adults consistent with the young age of the present case.

### 2.3. Case 3: Fever of Unknown Origin and Delayed Seroconversion

A 53-year-old man was referred with persistent fever and fatigue for seven days. Symptoms began with sudden fever and loss of appetite, without other complaints. An outpatient workup had revealed elevated liver enzymes and splenomegaly. Travel history included a trip to New Zealand within the past year, and his medical history included daily alcohol consumption.

On examination, a supraclavicular lymph node was palpable. Laboratory results showed markedly elevated CRP, but normal lymphocyte counts and liver enzymes were elevated (Table 4, and Supplemental Table S3). Serologies for common viruses causing hepatitis, including EBV, were negative. Over the following days, the laboratory revealed lymphocytosis with reactive morphology and increased large unstained cells (LUCs). Fever persisted with up to 40 °C.

CT imaging showed mediastinal, hilar, and axillary lymphadenopathy, hepatosplenomegaly (spleen diameter 14 cm), bilateral pleural effusions, and nonspecific pulmonary ground-glass opacities. Liver ultrasound revealed steatosis without focal lesions. Blood cultures and autoimmune panels were negative.

**Figure 2:**
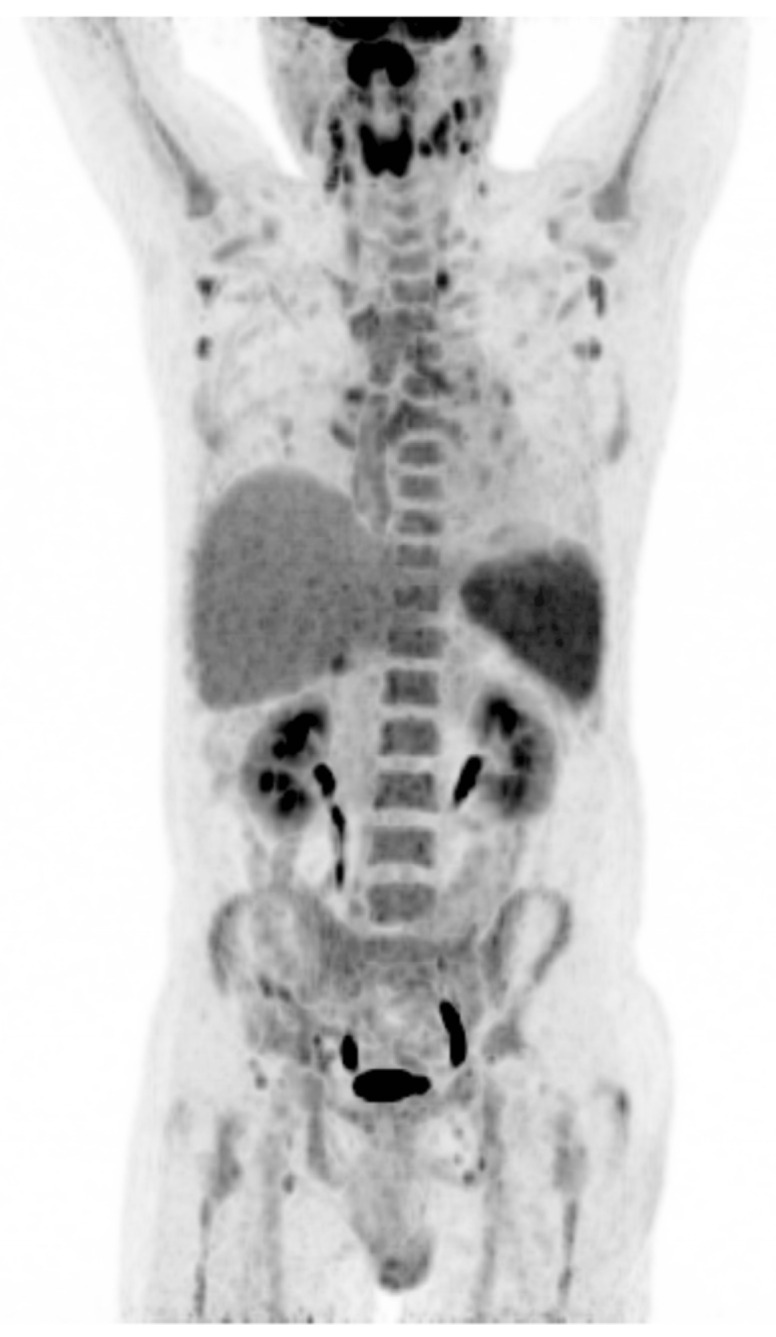
18F-FDG PET/CT scan (coronal maximum intensity projection) was ordered to evaluate fever of unknown origin. Intense uptake in the lymphatic tissue of the Waldeyer’s pharyngeal ring, the spleen, peripheral lymph nodes, and bone marrow are depicted consistent with a diagnosis of primary EBV infection in context of characteristic laboratory findings and not lymphoma. 18F-FDG = 18-fludeoxygluose; PET/CT = positron emission tomography/computed tomography.

Due to persistent fever of unknown origin, a 18F-FDG PET/CT scan was performed. This showed intensely metabolically active, enlarged supra- and infradiaphragmatic lymph nodes including intensely increased metabolism of the lymphatic tissue of the Waldeyer’s pharyngeal ring, a splenomegaly with diffusely increased metabolism, and evenly increased metabolism of the bone marrow. No further metabolically active malignant foci were delineated (Figure 2).

Despite repeated negative EBV serology, the clinical picture—fever, lymphadenopathy, lymphocytosis with large unstained cells (LUCs), splenomegaly, and mild hepatitis—was suggestive of IM. EBV PCR therefore was performed and returned positive, confirming primary EBV infection as cause of the symptoms. Simultaneously, the results of the FDG PET-CT scan were obtained, matching the findings in this context as reactive due to the EBV infection and not as evidence of a malignant disease, such as a lymphoma.

Inflammatory markers and liver enzymes gradually normalized; fever was resolved two weeks after onset. Notably, EBV seroconversion was delayed: both VCA-IgM and -IgG remained negative for up to 8 weeks (negative tested on day 12, day 20, and day 54 after symptoms onset). VCA-IgG and EBNA-1 only became detectable on a check-up 27 weeks later (positive on day 158 after symptoms onset, see Supplemental Table S3).

This case highlights the importance of clinical judgment in patients with strong clinical suspicion for IM and the importance of EBV PCR testing to establish a diagnosis of IM, particularly when serological testing is inconclusive or delayed. It also illustrates that late seroconversion may occur, potentially complicating diagnosis.

The cause for the late seroconversion is not understood. There are other reported yet asymptomatic cases with a delayed immune response of several months [7]. The median time to positivity for detection of VCA-antibodies after illness onset is 2 days for VCA-IgM and 31 days for VCA-IgG, based on prospective studies of EBV naïve college students in the USA [3].

### 2.4. Case 4: Secondary Hemophagocytic Lymphohistiocytosis Triggered by Primary EBV Infection

A 78-year-old man with persistent fever and elevated liver enzymes following a recent three-week trip to Thailand was referred to our hospital. Pronounced symptoms began shortly after his return and included fatigue, flu-like symptoms, and fever of up to 39 °C, although a general deterioration of his condition had already occurred during the travel. His medical history included hypertension (treated with perindopril/indapamide) and benign prostate hyperplasia.

Initially evaluated by an outpatient physician, he was managed symptomatically with paracetamol. Laboratory results were remarkable for an elevated CRP, mild thrombocytopenia and elevated liver enzymes. Due to persistent fever, worsening general condition and new icteric sclerae, he was admitted.

On admission, the patient remained febrile and jaundiced, though physical examination was otherwise unremarkable. Laboratory tests revealed cholestatic hepatitis with three to five times elevated liver enzymes and elevated bilirubin (Table 5 and Supplemental Table S4). Ultrasound showed no biliary obstruction. Inflammatory markers remained stable. While no lymphocytosis was present, reactive lymphocyte morphology was noted and thrombocytopenia persisted.

Given the patient’s recent travel to Thailand, several travel-associated infectious diseases were ruled out, and repeated blood cultures were negative. Persistent fever and constitutional symptoms prompted CT imaging, revealing hepatosplenomegaly (spleen volume 340 mL, diameter 13.8 cm) and enlarged mediastinal, hilar, axillary, and periportal lymph nodes. Due to regular contact with young grandchildren, serologic testing for EBV and CMV was performed. EBV serology showed positive VCA-IgM and -IgG, as well as positive EBV PCR with negative EBNA-1, confirming a diagnosis of primary EBV infection at the age of 78 years (Supplemental Table S4).

Concurrently, ferritin was markedly elevated (15,133 µg/L) alongside a progressive bicytopenia and persisting fever, prompting evaluation for hemophagocytic lymphohistiocytosis (HLH) triggered by primary EBV infection as the most likely diagnosis. An HScore of already 214 points indicated a 94% probability of HLH and diagnostic criteria of the HLH-2004 protocol were fulfilled (hyperferritinemia, fever, splenomegaly, elevated triglycerides and decreased fibrinogen and, subsequently, also severe pancytopenia). After discussion with our hematologists, a secondary HLH triggered by primary EBV infection was deemed most likely, even without confirmation from a bone or spleen biopsy. Given the patient’s rapid clinical deterioration during the following days (persistent fevers, fluctuating consciousness, respiratory distress, and progressive cytopenia and hyperferritinemia), high-dose dexamethasone (16 mg/day) was initiated which was supplemented by etoposide (see below).

HLH is a rare, life-threatening hyperinflammatory syndrome caused by uncontrolled activation of macrophages and cytotoxic T cells. It may be primary (genetic, mainly in infants and children) or secondary, often triggered by infections, malignancies, or autoimmune diseases. EBV infection has been reported to account for approximately 70% of infection-related HLH, especially in the Asian population [20,21,22].

The exact mechanism of EBV triggering secondary HLH is unknown, but it seems that EBV-infected CD8+ T cells and natural killer cells are critically involved, become overactivated and secrete large amounts of inflammatory cytokines causing a cytokine storm, ultimately leading to tissue destruction and organ failure in HLH [23].

In this case, secondary HLH was triggered by late-onset primary EBV infection. Hepatosplenomegaly and cholestatic hepatitis were attributed to lymphohistiocytic infiltration. The patient later developed confusion, and respiratory distress, requiring ICU transfer. Cerebrospinal fluid analysis revealed elevated protein counts, consistent with central nervous system involvement of HLH. Because of the rapid clinical deterioration with involvement of central nerval system, etoposide was added to therapy, along with broad-spectrum antibiotics for secondary infections caused by the severe pancytopenia attributed to HLH. Despite treatment, the patient developed progressive multi-organ failure and pancytopenia, resulting in death.

This case highlights that primary EBV infection, especially in elderly patients, may trigger severe secondary HLH. Early symptoms of EBV and HLH may overlap, complicating timely diagnosis. In returning travelers with fever and systemic symptoms, entertaining a wide differential diagnosis for infectious causes is essential. In this case, a primary EBV infection was not associated with travel but precipitated HLH, demonstrating that common pathogens may underlie rare, fatal syndromes. HLH remains a crucial differential diagnosis in febrile patients with cytopenia and organ dysfunction.

## 3. Discussion and Conclusions

Unusual presentations of primary EBV infections are challenging for the busy clinician/hospitalist and, hence, our case series serves as a reminder for the clinical and laboratory features of primary EBV infection and might help to adopt a stepwise approach toward diagnosis, avoiding over-testing. For example, if more extensive serologic testing is ordered in patients with acute EBV infection (e.g., CMV serology), there is a substantial risk for false positive serological results (particularly IgM) due to the polyclonal activation of B cells [24,25].

The cases illustrate the broad spectrum of presentations of primary EBV infection, thereby facilitating earlier diagnosis. The symptoms are variable and can present with variable degrees of severity. Regardless of patient age, EBV infection should be considered when there is lymphocytosis with reactive lymphocyte morphology, elevated large unstained cells, persistent fever, pharyngitis and lymphadenopathy, splenomegaly, and hepatitis—the latter typically being mild, but it may present as severe cholestatic hepatitis.

Acute hepatitis is seen in 75–80% of patients with IM, but the liver involvement is usually subclinical in 90–95% of the cases, while jaundice may only occur in 5% [26,27,28]. Liver enzymes are commonly 3–5 times the upper limit, whereas levels 10-fold and above are rare [27]. In case 1, the severe cholestatic hepatitis led to a hospital referral for further evaluation, where primary EBV infection was diagnosed. Despite the, in retrospect, characteristic symptoms of IM, cholestatic hepatitis may also occur in the absence of IM manifestations [29]. This case underscores the importance of maintaining awareness of rare manifestations of EBV, as it can potentially avoid hospital admissions.

Periorbital edema can precede more classical features of an IM, with an incidence of as high as 56% in a prospective study of 26 EBV-positive individuals, when specifically assessed, suggesting that it may be underrecognized [19]. Although the Hoagland sign is well documented in the medical literature for IM, awareness within routine clinical practice remains inadequate and may delay EBV testing. This can prompt unnecessary investigations or treatments, including antibiotics, as in case 2 [30]. Also, severe EBV pharyngitis can mimic streptococcal tonsillitis and may lead to inappropriate antibiotic treatment. This emphasizes the importance of an early recognition of EBV.

Important differential diagnoses with similar features are primary infections with the CMV and HIV. Whereas pharyngitis with or without tonsillar enlargement, tonsillar exudate, and cervical lymphadenitis are prominent features of primary EBV infection with or without a rash, symptomatic CMV infection often presents with only fever (often prolonged compared to EBV) in young to middle-aged adults (older age of onset compared to primary EBV infection), milder lymphadenopathy and splenomegaly, milder lymphocytosis with reactive lymphocytes, often rather low C-reactive protein concentrations (less than 100 mg/L) [28], and often no evidence of pharyngitis compared to primary EBV infection. In contrast, acute HIV infection may manifest with fever, pharyngitis, generalized (in contrast to localized cervical) lymphadenopathy and lymphopenia (plus/minus thrombocytopenia), and a maculopapular rash, but usually without relevant hepatitis. In unclear settings with typical features, these differential diagnoses need to be excluded. Depending on the presentation, malignancy such as lymphoma can also present with similar features of lymphadenopathy, hepatomegaly, and fever, and needs to be kept in consideration, as it was performed in case 3.

If strong clinical suspicion for EBV remains, even an initially negative serology should not lead to a premature closure of the case, and primary EBV infection should still be entertained in the differential diagnosis and actively sought by ordering an EBV PCR test. Data on frequency of delayed seroconversion in EBV primary infection is rare and has been described in asymptomatic patients (IgM/IgG VCA). In a study of 90 patients with symptoms of IM for 2 weeks or less and a positive VCA-IgM test, 29% showed no EBNA antibodies after 6 months follow-up (varying depending on the analytical method applied) [31]. A failure to develop EBNA antibodies is reported to be as high as 5–10% of patients who have been infected with EBV, as stated by the Infectious Diseases Society of America [32]. In a recent prospective study of university students developing primary EBV infection, a rapid and effective antibody response correlated with milder disease and earlier seroconversion, whereas delayed antibody responses were associated with more severe or prolonged symptoms and later seroconversion [33]. Interestingly, VCA-IgG seroconversion was detectable in all individuals with mild symptoms until day 100, whereas this was the case for only 60% of individuals with moderate/severe symptoms with delayed seroconversion up to day 200. This suggests that the kinetics of antibody production are influenced by the magnitude and timing of the host’s cellular immune response. Case 3 underscores these findings, where prolonged symptoms such as fever matched a late seroconversion, and an EBV PCR was necessary to establish the diagnosis of primary EBV infection. However, the exact immunological mechanisms of delayed seroconversion remain to be established.

If primary EBV infection is diagnosed, it can resemble the early clinical presentation of HLH, as illustrated in case 4. The prevalence of HLH varies among countries, with a reported prevalence for primary HLH of 1 in 50,000 live births in Sweden. The prevalence for secondary HLH is less well established and estimated to be, overall, 4.2 cases per 1 million population in England 2018 [12]. HLH in the elderly is far less frequent, usually of secondary origin, with malignancies and infections serving as main triggers (most common trigger is EBV).

Outcome for elderly people (50–87 years), as described by Altook 2019 [34] in a literature review of 71 cases, was dismal. In particular, if HLH was caused by a viral trigger, the mortality rate was found to be 84% [34].

Recognition of clinical features for HLH is key. They can mimic infections, fever of unknown origin, hepatitis, or encephalitis. Clinical features according to HLH-2004 protocol are fever, splenomegaly, bicytopenia, hypertriglyceridemia or hypofibrinogenemia, hemophagocytosis, hyperferritinemia (>500 µg/L), and elevated soluble CD25. Secondary HLH often manifests as critical illness with sepsis-like manifestations. Hence, as in case 4, if severity of the clinic presentation or the laboratory findings are not in line with a diagnosis of primary EBV infection, in particular, the presence of prolonged, high-grade fevers, evidence of central nervous system dysfunction, coagulopathy, severe cytopenias, and elevated ferritin concentrations above 5000–10,000 µg/L, HLH criteria should be actively assessed as secondary HLH is still underdiagnosed [12].

Treatment of primary EBV infection is usually symptomatic, with immunosuppressive treatment being required for secondary HLH. With the exception of case 4, all patients received supportive treatment with intravenous fluids and either acetaminophen or nonsteroidal anti-inflammatory drugs when primary EBV infection was finally confirmed. Case 2 also received antibiotic treatment due to initial concerns of cellulitis. As patients 1–3 had already improved when primary EBV infection was diagnosed and data regarding their efficacy are lacking, corticosteroids and, in particular, acyclovir were not used for treatment. Case 4 was treated with high-dose dexamethasone and etoposide as part of the HLH-94 protocol.

### Conclusions

Unusual presentations of primary EBV infection may complicate the diagnostic process and may lead to unnecessary testing. Our case series underscores the broad clinical spectrum of primary EBV infection and highlights key features that aid in distinguishing it from important differential diagnoses such as primary CMV infection or acute HIV. Awareness of characteristic laboratory findings, including reactive lymphocytosis, elevated large unstained cells, persistent fever, lymphadenopathy, splenomegaly, and mild-to-moderate hepatitis, is essential for guiding a targeted diagnostic approach for primary EBV infections while being vigilant for complications such as cholestatic hepatitis and HLH.

## Figures and Tables

**Table 1:** State-of-the-art interpretation of EBV-specific serological profiles for diagnoses.

Heterophilic Antibodies	Atypical Lymphocytes	VCA-IgG	VCA-IgM	EBNA-1 IgG	Interpretation
+/−	+	+	+	-	acute infection
-	-	+	-	+	past infection
-	-	-	-	-	no infection
+/−	+/−	+	-	-	indeterminate ^1,2^
-	-	+	+	+	indeterminate ^1,3^
-	+/−	-	+	-	indeterminate ^1,4^
-	-	-	-	+	not plausible

+ positive, − negative, ^1^ Re-assess clinical pre-test probability and potential differential diagnoses. For clarification of EBV infection, further testing is needed, such as PCR or repeat serology 2–4 weeks later. ^2^ past infection with failure of EBNA-1 seroconversion most likely; ^3^ past infection with false positive VCA-IgM most likely; ^4^ acute EBV infection possible, particularly in cases with presence of lymphocytosis and atypical lymphocytes. Adapted from Hess et al. [11].

**Table 2:** Laboratory results on referral and peak values of severe EBV induced cholestatic hepatitis.

Parameter	Unit	Reference Range	Case 1	
			On Referral	Peak ^1^
lymphocytes	G/L	1.17–3.45	4.2	7.7
LUC	G/L	<0.4	0.97	1.17
hemoglobin	g/L	123–158 (f)	147	n/a
thrombocytes	G/L	150–400	119	119
CRP	mg/L	<5	36	36
ASAT	U/L	<52	512	586
ALAT	U/L	<50	515	780
bilirubin	µmol/L	<20.5	102.8	108.3
ferritin	µg/L	22–275	n/t	885

^1^ peak of pathological value; LUC = large unstained cells, CRP = C-reactive protein, ASAT = aspartate aminotransferase, ALAT = alanine aminotransferase, n/t = not tested, n/a = not applicable, f = female.

**Table 3:** Laboratory results on referral and peak values for IM with Hoagland sign.

Parameter	Unit	Reference Range	Case 2	
			On Referral	Peak ^1^
lymphocytes	G/L	1.17–3.45	0.9	6.76
LUC	G/L	<0.4	0.24	12.82
hemoglobin	g/L	123–158 (f)	152	n/a
thrombocytes	G/L	150–400	219	n/a
CRP	mg/L	<5	10	12
ASAT	U/L	<52	24	487
ALAT	U/L	<50	25	1044
bilirubin	µmol/L	<20.5	3.3	n/a

^1^ peak of pathological value; LUC = large unstained cells, CRP = C-reactive protein, ASAT = aspartate aminotransferase, ALAT = alanine aminotransferase; n/a = not applicable, f = female.

**Table 4:** Laboratory results on referral and peak values for primary EBV infection manifesting as fever of unknown origin.

Parameter	Unit	Reference Range	Case 3	
			On Referral	Peak ^1^
lymphocytes	G/L	1.17–3.45	1.61	14.58
LUC	G/L	<0.4	0.39	2.05
hemoglobin	g/L	139–165 (m)	147	n/a
thrombocytes	G/L	150–400	282	n/a
CRP	mg/L	<5	173	173
ASAT	U/L	<52	189	315
ALAT	U/L	<50	177	337
bilirubin	µmol/L	<20.5	16.9	n/a
ferritin	µg/L	22–275	n/t	5094

^1^ peak of pathological value; LUC = large unstained cells, CRP = C-reactive protein, ASAT = aspartate aminotransferase, ALAT = alanine aminotransferase, n/t = not tested, n/a = not applicable, m = male.

**Table 5:** Laboratory results on referral and peak values for primary EBV infection and secondary HLH.

Parameter	Unit	Reference Range	Case 4	
			On Referral	Peak ^1^
lymphocytes	G/L	1.17–3.45	2.79	9.78
LUC	G/L	<0.4	1.40	1.40
hemoglobin	g/L	139–165 (m)	142	68
thrombocytes	G/L	150–400	128	4 ^2^
CRP	mg/L	<5	49	245
ASAT	U/L	<52	156	347
ALAT	U/L	<50	151	192
bilirubin	µmol/L	<20.5	39.2	158
ferritin	µg/L	22–275	n/t	41,308

^1^ peak of pathological value. ^2^ nadir value for platelets; LUC = large unstained cells, CRP = C-reactive protein, ASAT = aspartate aminotransferase, ALAT = alanine aminotransferase, n/t = not tested, m = male.

## Data Availability

The original contributions presented in this study are included in the article. Further inquiries can be directed to the corresponding author.

## Supplementary Materials

The following supporting information can be downloaded at: https://www.mdpi.com/article/10.3390/jcm14248747/s1, Table S1: Case 1, laboratory results. Abbreviations: LUC = large unstained cells, CRP = C-reactive protein, ASAT = aspartate aminotransferase, ALAT = alanine aminotransferase, GGT = Gamma glutamyltransferase, n/t = not tested; f = female; S/CO = sample-to-cut-off, a S/CO of >1 is a positive test result; Table S2: Case 2, laboratory results. Abbreviations: LUC = large unstained cells, CRP = C-reactive protein, ASAT = aspartate aminotransferase, ALAT = alanine aminotransferase, GGT = Gamma glutamyltransferase, n/t = not tested; f = female; S/CO = sample-to-cut-off, a S/CO of >1 is a positive test result; Table S3: Case 3, laboratory results. Abbreviations: LUC = large unstained cells, CRP = C-reactive protein, ASAT = aspartate aminotransferase, ALAT = alanine aminotransferase, GGT = Gamma glutamyltransferase, n/t = not tested; m = male; S/CO = sample-to-cut-off, a S/CO of >1 is a positive test result; Table S4: Case 4, laboratory results. Abbreviations: LUC = large unstained cells, CRP = C-reactive protein, ASAT = aspartate aminotransferase, ALAT = alanine aminotransferase, GGT = Gamma glutamyltransferase, n/t = not tested; m = male; S/CO = sample-to-cut-off, a S/CO of >1 is a positive test result.

## Abbreviations

The following abbreviations are used in this manuscript:

ALAT	alanine aminotransferase
ANA	antinuclear antibodies
ASAT	aspartate aminotransferase
CMV	Cytomegalovirus
CRP	C-reactive protein
CT	computer tomogram
EBNA-1	EBV nuclear antigen 1
EBV	Epstein–Barr virus
GGT	Gamma glutamyltransferase
HHV-4	Human Herpesvirus 4
HIV	human immunodeficiency virus
HLH	hemophagocytic lymphohistiocytosis
IgG	immunoglobulin G
IgM	immunoglobulin M
IM	Infectious mononucleosis
LUC	large unstained cells
PCR	polymerase chain reaction
pEVB	Primary Epstein–Barr virus infection
VCA	virus capsid antigen
18F-FDG PET	18F-Fluordesoxyglucose Positronenemissionstomographie
