# Supplementary material for: The Many Faces of Primary EBV Infection: A Case Series of Its Diverse Presentations
Source: J Clin Med. 2025 Dec 10;14(24):8747. doi: 10.3390/jcm14248747 (PMC12734239; doi:10.3390/jcm14248747)
Supplement: Supplementary file 1 [file jcm-14-08747-s001.zip › jcm-4003440-supplementary.pdf]

Journal of
Clinical Medicine

MDPI

Supplementary Materials

Table S1. Case 1, laboratory results.

parameter	unit	reference range			
**days after symptoms on-set**			**7**	**8**	**10**
			**on referral**		
hemoglobin	g/l	123–158 (f)	147	123	129
thrombocytes	G/l	150–400	119	133	165
leukocytes	G/l	3.0–9.6	8.15	9.23	11.19
lymphocytes	G/l	1.17–3.45	4.2	5.85	7.7
LUC	G/l	<0.4	0.97	1.09	1.17
CRP	mg/l	<5	36	27	13
Ferritin	µg/l	22–275	885	n/t	n/t
bilirubin	µmol/l	<20.5	102.8	95.3	108.3
ASAT	U/l	<52	512	586	432
ALAT	U/l	<50	515	693	780
GGT	U/l	<36	167	133	107
EBV DNA PCR	IE/ml				1080
EBV IgM	S/CO		0.82 (borderline)		2.12 (positive)
EBV VCA IgG	S/CO		0.31 (negative)		
EBV EBNA-1 IgG	S/CO		0.01 (negative)		

LUC = large unstained cells, CRP = C-reactive protein, ASAT = aspartate aminotransferase, ALAT = alanine aminotransferase, GGT = Gamma glutamyl-transferase, n/t = not tested; f = female; S/CO = sample-to-cut-off, a S/CO of >1 is a positive test result.

Table S2. Case 2, laboratory results.

parameter	unit	reference range							
**days after symptoms on-set**			**2**	**3**	**6**	**9**	**10**	**13**	**17**
			**on refer-ral**						
hemoglobin	g/l	123–158 (f)	152	151	154	158	149	143	143
thrombocytes	G/l	150–400	219	203	188	211	231	320	321
leukocytes	G/l	3.0–9.6	4.65	5.5	7.54	10.55	12.82	12.69	11.41
lymphocytes	G/l	1.17–3.45	0.88	2.24	3.63	5.57	6.55	6.76	5.05
LUC	G/l	<0.4	0.24	n/t	0.85	1.23	1.76	0.99	0.91
CRP	mg/l	<5	10	12	5	4	1	n/t	n/t
Ferritin	µg/l	22–275	n/t	n/t	n/t	n/t	620	n/t	n/t
bilirubin	µmol/l	<20.5	3.3	n/t	5.1	6.8	n/t	n/t	n/t
ASAT	U/l	<52	24	n/t	340	347	338	68	36
ALAT	U/l	<50	25	n/t	358	1044	935	494	191
EBV IgM	S/CO				0.80 (borderline)			8.53 (positive)	
EBV VCA IgG	S/CO				0.42 (negative)			2.17 (positive)	
EBV EBNA-1 IgG	S/CO				0.06 (negative)			0.06 (negative)	

LUC = large unstained cells, CRP = C-reactive protein, ASAT = aspartate aminotransferase, ALAT = alanine aminotransferase, GGT = Gamma glutamyl-transferase, n/t = not tested; f = female; S/CO = sample-to-cut-off, a S/CO of >1 is a positive test result.

Table S3. Case 3, laboratory results.

parameter	unit	reference range										
**days after symptoms onset**			**7**	**11**	**12**	**14**	**16**	**20**	**24**	**30**	**54**	**158**
			**on re-ferral**									
hemoglobin	g/l	139–165 (m)	147	134	142	130	148	144	154	144	142	157
thrombo-cytes	G/l	150–400	282	332	353	370	398	362	458	404	444	377
leukocytes	G/l	3.0–9.6	5.88	13.19	19.51	19.01	22.06	8.15	10.73	9.31	6.84	5.19
lymphocytes	G/l	1.17–3.45	1.61	7.51	11.1	12.41	14.58	3.63	6.96	6.3	4.28	2.59
LUC	G/l	<0.4	0.39	1.37	2.04	1.66	2.05	0.31	0.48	0.34	0.22	0.17
CRP	mg/l	<5	173	103	74	38	37	71	15	n/t	n/t	n/t
Ferritin	µg/l	22–275	n/t	n/t	5094	4017	n/t	3707	n/t	n/t	n/t	n/t
bilirubin	µmol/l	<20.5	16.9	15.8	19	n/t	12	18.1	13.8	n/t	n/t	n/t
ASAT	U/l	<52	189	315	268	130	128	129	90	n/t	43	n/t
ALAT	U/l	<50	177	337	332	191	150	123	110	n/t	52	n/t
GGT	U/l	<36	n/t	n/t	442	44	579	456	370	n/t	n/t	n/t
EBV DNA PCR	IE/ml					3350						
EBV IgM	S/CO				0.24 (negative)			0.18 (negative)			0.01 (neg.)	0.35 (neg.)
EBV VCA IgG	S/CO				0.04 (negative)			0.07 (negative)			0.20 (neg.)	86.2 (pos.)
EBV EBNA-1 IgG	S/CO				0.01 (negative)			0.01 (negative)			0.01 (neg.)	7.09 (pos.)

LUC = large unstained cells, CRP = C-reactive protein, ASAT = aspartate aminotransferase, ALAT = alanine aminotransferase, GGT = Gamma glutamyl-transferase, n/t = not tested; m = male; S/CO = sample-to-cut-off, a S/CO of >1 is a positive test result.

Table S4. Case 4, laboratory results.

parameter	unit	reference range												
**days after symptoms onset**			**8**	**9**	**10**	**11**	**13**	**16**	**19**	**22**	**25**	**28**	**31**	**34**
			**on re-ferral**											
hemoglobin	g/l	139–165 (m)	142	130	128	122	123	113	104	96	81	74	73	86
thrombo-cytes	G/l	150–400	128	109	101	107	108	123	110	69	74	55	10	4
leukocytes	G/l	3.0–9.6	9.78	6.07	6.41	7.05	5.51	5.75	5.91	3.96	1.86	0.39	0.04	0.05
lymphocytes	G/l	1.17–3.45	2.79	1.48	1.84	2.61	1.58	1.78	1.57	0.74	0.26	0.1	0.04	0.05
LUC	G/l	<0.4	1.4	0.8	1.06	1.18	0.62	0.74	0.54	0.2	0.02	0.03	n/t	n/t
CRP	mg/l	<5	49	n/t	n/t	62	64	45	66	28	55	55	245	120
Ferritin	µg/l	22–275	n/t	n/t	15133	n/t	16880	12643	10398	n/t	34958	n/t	27483	41308
triglyceride	mmol/l	<1.7	n/t	n/t	n/t	3.6	2.7	3.4	n/t	n/t	n/t	n/t	5.7	n/t
bilirubin	µmol/l	<20.5	39.2	40.8	51.8	62.4	78.6	115.7	n/t	111.6	116.4	109.8	134.5	158.3
ASAT	U/l	<52	156	140	161	n/t	168	174	n/t	n/t	211	199	179	347
ALAT	U/l	<50	151	128	131	127	114	110	n/t	n/t	119	142	130	192
GGT	U/l	<36	755	591	586	n/t	n/t	n/t	n/t	n/t	n/t	n/t	n/t	n/t
EBV DNA PCR	IE/ml					49800								
EBV IgM	S/CO					42.3 (positive)								
EBV VCA IgG	S/CO					1.21 (positive)			1.0 (positive)					
EBV EBNA-1 IgG	S/CO					0.01 (negative)								

LUC = large unstained cells, CRP = C-reactive protein, ASAT = aspartate aminotransferase, ALAT = alanine aminotransferase, GGT = Gamma glutamyl-transferase, n/t = not tested; m = male; S/CO = sample-to-cut-off, a S/CO of >1 is a positive test result.
